# PCSK9 Promotes Cardiovascular Diseases: Recent Evidence about Its Association with Platelet Activation-Induced Myocardial Infarction

**DOI:** 10.3390/life12020190

**Published:** 2022-01-27

**Authors:** Meidi Utami Puteri, Nuriza Ulul Azmi, Mitsuyasu Kato, Fadlina Chany Saputri

**Affiliations:** 1 Department of Pharmacology-Toxicology, Faculty of Pharmacy, Universitas Indonesia, Depok 16424, Indonesia; meidiutami@farmasi.ui.ac.id (M.U.P.); nuriza.azmi@farmasi.ui.ac.id (N.U.A.); 2 Ph.D. Program in Humanics, School of Integrative and Global Majors, University of Tsukuba, Tsukuba 305-8575, Japan; 3 Department of Experimental Pathology, Faculty of Medicine, University of Tsukuba, Tsukuba 305-8575, Japan; mit-kato@md.tsukuba.ac.jp

**Keywords:** atherogenesis, atherosclerosis, atherothrombosis, cardiovascular disease, cluster of differentiation 36, myocardial infarction, proprotein convertase subtilisin/kexin type 9, platelet activation

## Abstract

Cardiovascular diseases are the leading cause of death worldwide, with the majority of the cases being heart failure due to myocardial infarction. Research on cardiovascular diseases is currently underway, particularly on atherosclerosis prevention, to reduce the risk of myocardial infarction. Proprotein convertase subtilisin/kexin type 9 (PCSK9) has been reported to play a role in lipid metabolism, by enhancing low-density lipoprotein (LDL) receptor degradation. Therefore, PCSK9 inhibitors have been developed and found to successfully decrease LDL plasma levels. Recent experimental studies have also implicated PCSK9 in platelet activation, having a key role during atherosclerosis progression. Although numerous studies have addressed the role of PCSK9 role in controlling hypercholesterolemia, studies and discussions exploring its involvement in platelet activation are still limited. Hence, here, we address our current understanding of the pathophysiological process involved in atherosclerosis-induced myocardial infarction (MI) through platelet activation and highlight the molecular mechanisms used by PCSK9 in regulating platelet activation. Undoubtedly, a deeper understanding of the relationship between platelet activation and the underlying molecular mechanisms of PCSK9 in the context of MI progression will provide a new strategy for developing drugs that selectively inhibit the most relevant pathways in cardiovascular disease progression.

## 1. Introduction

According to recent data from the Global Burden of Disease (GBD), cardiovascular diseases are still the leading cause of disease burden worldwide [1]. Their prevalence, morbidity, and mortality have increased in 204 countries and territories from 1990 to 2019 [2]. Moreover, an analysis study by the American Heart Association predicted that the total costs of cardiovascular disease burden will increase up to $1.1 trillion in 2035, making cardiovascular diseases the most costly among chronic diseases [3]. Data from the World Health Organization (WHO) also noted cardiovascular diseases as the number one cause of death worldwide, with an estimated 17.9 million deaths in 2019, accounting for 32% of all global deaths [4]. Cardiovascular diseases are a group of diseases related to heart and blood vessel disorders, including peripheral artery disease, cerebrovascular disease, and coronary artery disease [5]. Coronary artery disease accounts for 30–50% of the total cardiovascular disease cases [4,6]. Coronary artery disease is caused by a low supply of oxygen-rich blood through the heart muscles, resulting in advanced cardiovascular incidents, such as myocardial infarction (MI) or even death [7,8]. It is usually indicated by an accumulation of atherosclerotic plaque in the arterial walls, which is commonly initiated by a hypercholesteremia condition [8]. Urgent interventions are required to minimize the burden of cardiovascular disease, including research on the prevention and treatment of atherosclerosis.

Proprotein convertase subtilisin/kexin type 9 (PCSK9), initially known as neural apoptosis-regulated convertase 1 (NARC-1), was identified as a new member of the PC family [9]. After the report about its roles in cholesterol metabolism, extensive studies were conducted to elucidate the association between PCSK9 and cardiovascular disease, as well as their risk factors [10,11]. Next, growing evidence has shown that PCSK9 involvement is a key factor in controlling plasma cholesterol levels, by enhancing the degradation of low-density lipoprotein receptors (LDLR) [12,13,14]. Moreover, recent studies have reported the other functions of PCSK9 in cardiovascular events, independently of LDL-cholesterol regulation, including its role in promoting platelet activation and coagulation during cardiovascular disease progression [15,16,17]. This makes PCSK9 a potential target to be developed for the prevention and treatment of cardiovascular diseases. Accordingly, PCSK9 inhibitors were established and found to improve cardiac function in an acute myocardial infarction (AMI) rat model [18].

While numerous studies have elucidated the roles of PCSK9 in altering LDL cholesterol plasma levels, via the PCSK9–LDLR axis, studies on its association with platelet activation-induced myocardial infarction (MI) are still very limited. Therefore, in this study, we provide a comprehensive discussion of PCSK9’s potential role in inducing myocardial infraction by promoting platelet activation, bringing novel insights to aid in the development of a better therapeutic MI treatment.

## 2. Myocardial Infarction (MI)

MI is defined as myocardial cell death caused by inadequate oxygen supply (ischemia) and is usually diagnosed based on the patient’s clinical presentation, medical record, and electrocardiogram (ECG) evaluation [19,20]. Cardiac troponin (cTn) levels are also used as biomarkers to check whether heart muscle has been damaged (myocardial injury) [19]. Various combinations of chest pain, epigastric discomfort, exhaustion, shortness of breath, and fatigue are possible ischemic symptoms [19,21]. According to the fourth universal definition of MI, myocardial injury detected by an abnormal value of cTn, together with the clinical presentation of myocardial ischemia should be categorized as MI [19]. To date, there are no clinically approved medicines as therapeutic agents for infarcted myocardial tissue regeneration [22,23]. Therefore, the main objective of MI therapy is to improve blood flow to the heart muscle (myocardial revascularization) and, to the greatest extent possible, slow the disease progression [22,23].

MI is divided into various types based on their pathological aspects [19]. MI type 1 is distinguished by atherosclerosis plaque disruption (erosion or rupture), which leads to atherothrombosis, and MI type 2 is characterized by myocardial injury followed by an ischemic condition due to lack of oxygen supply, without any features of atherothrombosis [19]. Practically, it is common to categorize MI as ST-elevation MI (STEMI) in patients who develop ST-segment elevations and ischemic symptoms [19]. Patients who do not have ST-segment elevation at the time of presentation, on the other hand, are considered to have non-ST-elevation MI (NSTEMI) [19]. Patients with STEMI and NSTEMI are categorized as part of acute coronary syndrome (ACS) [19].

Platelet activation and the coagulation cascade are critical in the onset and progression of MI [22,24]. As a result, adequate platelet inhibition and anticoagulation are required for MI treatment, particularly for those undergoing myocardial revascularization via invasive treatments [22,24]. In an emergency, both STEMI and NSTEMI patients can be administered chewable aspirin right away [20]. If less oxygen saturation is observed, the patient should be given intravenous access and oxygen supplementation [20,23]. To alleviate chest pain and oxygen deprivation, opioids and nitroglycerin may be used, respectively [20,23]. Immediate myocardial revascularization is part of STEMI treatment [20,23]. In addition, percutaneous coronary intervention (PCI) is the preferred management therapy [20,23]. Before undergoing PCI treatment, the patient is given dual antiplatelet medicines [20,23]. Aspirin or intravenous heparin, in combination with a potent P2Y12 receptor inhibitor (ticagrelor or prasugrel), is the gold standard of therapy [20,23]. Inhibitors of glycoprotein IIb/IIIa or direct thrombin may also be used [20,23]. In NSTEMI patients, antithrombotic drugs must be administered with and without myocardial revascularization invasive treatment [22]. Notably, in the selection of drugs, the patient’s risk of ischemia and bleeding should be taken into account [22]. To achieve disease stabilization in patients diagnosed after an acute MI, lifestyle modifications (i.e., eating a healthy diet, regular exercising, reduction of body weight, and stopping smoking and drinking alcohol) and pharmacological therapies (i.e., antihypercholesterolemia, antihypertensive, and antiplatelet medications) are used [21,22]. However, when compared to treatment, prevention therapy remains the best option. Therefore, finding a strategy to slow down the progression of atherosclerosis should pave the way for a new approach to treating MI. This includes finding a new molecular target that is more effective and powerful against atherosclerosis.

## 3. Roles of Platelets during Atherosclerosis-Induced MI

### 3.1. Atherosclerosis

Hypertension, hypercholesterolemia, diabetes, obesity, an unhealthy diet, and a lack of exercise are all major risk factors for cardiovascular disease [25]. These risk factors are known to be related to atherosclerosis, a hallmark of almost all cardiovascular diseases [24]. Atherosclerosis is a condition in which arteries become narrow and stiff due to filling up with the plaque that results from the deposition of lipid molecules inside the arterial walls [26]. It starts with the infiltration, entrance, and retention of lipid molecules, particularly LDLs, into the intima of the arterial walls [26]. Once it is sequestered in the artery intima, LDL particles tend to have modifications such as aggregation, oxidation, cleavage, and incorporation with the immune complex to make LDLs become pro-atherogenic molecules [26]. The pro atherogenic LDLs, or so-called oxidized LDL (oxLDL), then induce the recruitment of monocytes and lymphocytes into the intima, which stimulates the differentiation of monocytes into macrophages that express scavenger receptors [24]. These receptors are known to be responsible for the uptake of cholesterol molecules and cholesterol esters into oxLDL, making macrophages become foam cells, the major characteristic of atherosclerotic lesions [24,27]. Notably, scavenger receptor class A (SRA), cluster of differentiation 36 (CD36), and lectin-like oxLDL receptor-1 (LOX-1) have been reported to have functions in oxLDL internalization that are essential for the formation of foam cells [24,27,28]. Once foam cells are generated, macrophage-derived foam cells secrete several chemokines that mediate sustained inflammatory response, leading to vascular remodeling, and increasing the chance of plaque disruption [24]. When the plaque is disrupted, which is then complemented with platelet activation and aggregation, the coagulation signaling pathways are activated to initiate the thrombosis or so-called acute atherothrombosis [24]. Moreover, LOX-1 expression on platelets also acts as an adhesion molecule to induce platelet aggregation by platelet agonist, adenosine diphosphate (ADP), and supports acute atherothrombosis [27,29]. Acute atherothrombosis results in solid clot formation inside the arterial walls, which restricts the supply of oxygen to the heart muscle and leads to ischemia, which is a major cause of AMI-induced death [24].

### 3.2. Roles of Platelets during Atherosclerosis Initiation

Platelets do not attach to endothelial cells in the arterial walls under normal physiological conditions [30]. However, endothelial lesion-induced inflammation has been shown to stimulate platelet attachment to endothelial cells [30]. In spite of this, one study has suggested that even if no endothelial lesions are detected, platelets could still be intact and adhere at lesion-prone sites on endothelial cells, as in the case of carotid artery bifurcation [30]. The attachment between the platelet and endothelial cells is reported to be mainly mediated by P-selectin, a cell adhesion molecule that is expressed on both the platelet and endothelial cells [24,30,31]. It starts with platelet tethering, the first and very brief contact between the platelet and endothelial cells that, in turn, activates the platelet and endothelial cells [31]. Next, the platelets adhere to the endothelial cells and enable firm adhesion, mediated by integrin binding [24,31]. The interaction between platelet GPIbα and αIIbβ3 (GPIIb/IIIa) with endothelial P-selectin has also been reported to be essential for platelet attachment to the endothelium [30]. 

### 3.3. Roles of Platelets during Atherosclerosis Progression (Atherogenesis)

In the majority of the cases in which atherosclerosis evolves to MI, there are two key steps that occur during the disease progression [24]. First is atherogenesis, and second is atherothrombosis, with platelet activation being involved in both processes [24]. It has been reported that during atherogenesis, platelet activation mediates the inflammatory response that enhances atherosclerotic plaque formation. Activated platelets are known to release chemokines, growth factors, coagulation protein, and pro-adhesion molecules that play essential roles in cell survival, proliferation, adhesion, coagulation, and proteolysis, all of which promote plaque formation [24,30]. Both the endothelial cells and activated platelets have also been reported to secrete cytokines such as interleukin 1 beta (IL-1β) and CD40L, which are known as pro-inflammatory factors that can stimulate nuclear factor-B (NFκB) pathway activation [30]. NFκB activation, next, stimulates the expression of essential genes that facilitate monocyte attachment and transmigration into the endothelium-adhered platelets, resulting in the acceleration of atherosclerotic plaque formation [32]. Ligand-CD36 binding in platelets is also known to activate multiple signaling pathways, such as the Src family kinases, mitogen-activated protein kinase (MAPK), and NADPH oxidase 2 (NOX2), all of which increase the generation of reactive oxygen species (ROS), known to be capable of stimulating platelet activation [33]. In addition, platelets also contribute to atherogenesis by facilitating oxLDL cholesterol intake to the arterial walls, for the formation of foam cells [27]. OxLDL is known to be taken up by SRA, CD36, and LOX-1 [24].

### 3.4. Roles of Platelets during Atherosclerosis Aggravation (Atherothrombosis)

Platelets are known to play an important role in the formation of thrombus following the rupture of an atherosclerotic plaque during atherosclerosis aggravation [24]. After the occurrence of vascular rupture, the subsequent step is the activation of the coagulation cascades that stimulate thrombus generation, which results in the conversion of fibrinogen to fibrin, stabilizing the platelet–thrombus aggregation, and finally, generating a solid clot [24]. Once the vascular wall is ruptured and makes a lesion, the extracellular matrix (ECM) components, such as fibronectin, laminin, and collagen become exposed to the blood component and release pro-inflammatory markers and cytokines, which lead to the adhesion of more platelets at the defect lesion site [24]. The platelets that have adhered then go through some changes, which causes them to secrete their cytoplasmic granules, including thromboxane (Tx) A2 and ADP [24,34]. They also go through a shape conformation change, which causes them to release various chemokines. It has also been reported that P2Y1 receptors are involved in the platelet conformational changes [24,35]. As the platelets become activated and adhere to each other, damage to the endothelial surface takes place, leading to the formation of a thrombus at the lesion site [24,35]. Stiff platelet and collagen adhesion also trigger platelet activation, which in turn results in a sustained and enhanced thrombotic process [24,35]. Thrombus generation at the plaque disruption site results from platelet binding to collagen via the GPVI receptor, which stimulates the activation of other platelet-adhesion receptors, such as integrins αIIbβ3 and α2β1 that promote solid, stable, and irreversible adhesion to the lesion surface [24,34]. Moreover, platelet CD36 signaling is known to activate cytosolic phospholipase A2 (cPLA2) via the p38MAPK pathway [36]. cPLA2 stimulates the release of arachidonic acid from membrane phospholipids, providing cyclooxygenase (COX)-1 to be converted into TXA_2_ [36]. TXA_2_ then works together with the downstream pathway to activate integrin αIIbβ3 [36].

To summarize, activated platelets are involved in a positive feedback loop that enhances and sustains the responses of platelets to the first stimulus, resulting in the high affinity of platelet binding. Collectively, this suggests the essential roles of platelet activation in atherosclerosis-induced MI, through both atherogenesis and atherothrombosis. Therefore, inhibiting platelet activation, as well as platelet coagulation, would be beneficial in preventing or slowing the progression of cardiovascular disease. However, the major side effects of the currently available antiplatelet and anticoagulant drugs are severe bleeding [37]. The unmet need in cardiovascular medicine research is the development of better, safer, and more effective drugs for the prevention and treatment of atherosclerosis-induced MI. Searching for new pathways by using molecularly targeted therapies is required to achieve this goal.

## 4. PCSK9 Contribution in Cardiovascular Events

### 4.1. The Discoveries of PCSK9

Many proteins are initially synthesized in an inactive form, or as a so-called precursor, as they contain amino acid chains that function to block their activity [38]. PCs cleave those chains to form active products from their original inactive form [38]. In 1998, the first proteinase properties in mammalian cells were observed, based on a study about the generation of human insulin, which is derived from its inactive precursor proinsulin [39]. They were further identified as the first two members of the PC family (types 1 and 2), currently known as PCSK1 and PCSK2 [40]. Later on, six members of the PC family were constitutively identified [40], namely furin, PCSK4, PCSK5, PCSK6, PCSK7, and subtilisin-kexin isozyme 1 (SKI-1) or membrane-bound transcription peptidase site 1 (MBTPS1) [40]. In 2002, a new cDNA, whose sequence is 24–25% similar to that of SKI-1 and PCSK7 was cloned and further identified in patented databases registered by Millennium Pharmaceuticals [40]. They obtained the sequence during their investigation of the serum inadequacy in primary cerebellar neurons that results in cells apoptosis [40]. Therefore, the gene was first named as neural apoptosis regulated convertase 1 (NARC-1) [40]; then, Seidah et al., identified it as the ninth member of the PCSK family and named it PCSK9 [9]. Without a conception of the enzyme’s function, Seidah’s research group continued to explore and reported its tissue and cellular distribution [9]. It was found to be highly expressed in the small intestine, liver, cortex, cerebellum, and kidney [9,40]. It was also revealed to be expressed in several tumor cell lines [9,40].

Transcriptional factors containing conserved sterol regulatory element (SRE) motif have been reported to be involved in modulating the gene expression that controls cholesterol metabolism [11]. It is the binding site of SRE-binding proteins (SREBPs), master regulators in lipid biosynthesis pathways [11]. Soon after the discovery of PCSK9, experimental approaches were employed to investigate the potential roles of SREBP in regulating PCSK9 gene expression. Using a microarray analysis in the hepatic mouse model, a study by Maxwell et al. demonstrated the upregulation of PCSK9 mRNA level in mice that overexpressed SREBP-1a or SREBP-2, as well as the downregulation of PCSK9 mRNA level in mice with cholesterol diet [41]. Interestingly, an in vitro study by Dubuc et al. found that PCSK9 expression was significantly induced by statin, 3-hydroxy-3-methylglutaryl coenzyme A (HMG-CoA) reductase inhibitors, in human primary hepatocytes and HepG2 cells, whereas the induction was cancelled by the addition of mevalonate [42]. This suggests a potential feedback loop of SREBP-2 activation, which results in increasing the mRNA level of PCSK9 [42]. Moreover, a specific binding between SREBP-1 and SREBP-2, and PCSK9 gene promoter SRE in vitro has been reported [11].

The human PCSK9 gene was found to be located at the human chromosome 1p32, with a size of 22-kb and consisting of 12 exons and 11 introns [35]. The PCSK9 gene encodes a 692-amino acid protein that is synthesized in the endoplasmic reticulum (ER) [35]. Similar to other PCSK9 family members, PCSK9, a membrane protease, consists of an amino-terminal signal peptide (SP), a pro-domain, and a subtilisin-like catalytic domain (SCD) [35,40]. Experimental studies, both in vitro and in vivo, and which were supported by clinical studies, demonstrated that enzymes belonging to the PCSK family have unique physiological functions, as they are involved in the regulation of numerous proteins to determine their inactivation or activation [35,40]. The first eight enzymes of the PCSK family, PCSK1, PCSK2, Furin, PCSK4, PCSK5, PCSK6, PCSK7, and SKI-1, are known to secrete their functions by cleave precursor protein (e.g., peptides and hormones), to generate active products that mature and play a role in cell metabolism [35,40]. On the other hand, PCSK9 does not function as a protease because PCSK9 cleaves itself, which makes it an outlier compared with other PCSK family members [35,40]. PCSK9 exerts its function in a non-enzymatic manner, to enhance the lysosomal and endosomal degradation of the major receptor involved in the LDL-c metabolism, the LDLR [35]. Hence, PCSK9’s catalytic activity is not required for its function on LDLR cycling [35]. Notably, the c-terminal domain of each PCSK member carries different sequences regulating their cellular trafficking and localization [40]. PCSK9 consists of a Cys-His-rich domain (CHRD) that later was found to be important for its interaction with LDLR [40].

### 4.2. PCSK9 and LDL Cholesterol Metabolism

The first report about the physiological functions of PCSK9 was its implication in liver regeneration and cortical neuron differentiation [9]. During a separate investigation, it was found that particular mutations in the PCSK9 gene result in autosomal dominant hypercholesterolemia [12]. The association between PCSK9 and hypercholesterolemia attracted numerous research groups to start an extensive investigation, resulting in the discovery moving from bench lab to clinical field in less than 10 years [40]. The significance of PCSK9 for LDL cholesterol homeostasis is demonstrated with gain- and loss-of-function mutations in PCSK9, which results in hyper- or hypocholesterolemia in individuals, respectively, with significant effects on atherosclerotic cardiovascular disease and further advanced incidence [12,35,43]. The molecular mechanisms underlying the PCSK9-LDLR interaction, which control lipid metabolism, have been well-reviewed [11,40,44]. In short, the PCSK9 c-terminal CHRD domain binds to the EGF-A repeat domain of LDLR and then targets it for intracellular degradation on the cell surface, resulting in a reduced number of LDL receptors on the cell surface and decreased elimination of LDL-cholesterol (LDL-c), which leads to enhance LDL-c in plasma [44,45,46].

Furthermore, in vivo studies using PCSK9 knockout mice have been established and demonstrated that the elimination of PCSK9 showed the phenotype of hypocholesterolemia, with an estimated 80% reduction in LDL-c, a strong decrease in the atherosclerosis development, and a significantly increased sensitivity to statin treatment [47,48], thus making PCSK9 as an interesting target for LDL-lowering therapies. Indeed, several approaches targeting PCSK9 have been developed, forming monoclonal antibodies, small peptide inhibitors, small interfering RNA, and gene silencing mediated by CRISPR/Cas9 [40]. In 2015, the US Food and Drug Administration (FDA) approved the first two PCSK9 mAB, namely, alirocumab and evolocumab. Several randomized clinical trials have demonstrated that these treatments successfully decreased LDL-c levels by 50–60% and increased high-density lipoprotein (HDL) cholesterol in patients with familial hypercholesterolemia and intolerance to statins, or those with a major risk of cardiovascular disease but unable to control their LDL-c levels with statins or ezetimibe [49].

### 4.3. PCSK9 and MI

Hyperlipidemia/hypercholesterolemia has been indicated as the major risk factor for MI [50]. Previous studies have reported the association between the progression of MI and serum lipid metabolism, whereas PCSK9 was found to be implicated in various physiological and pathological factors for lipid metabolism [14,51]. The link between PCSK9 and MI started to gain attention as several studies demonstrated a strong relationship between them. A cross-sectional study directed by Almontashiri et al. revealed that during AMI, the PCSK9 plasma level was enhanced in individuals (non-diabetic) with angiographically-defined coronary artery disease [52]. The individuals were limited to those who were not consuming any lipid-lowering medications [52]. A large prospective population study of individuals in Norway, by Laugsand et al., also demonstrated that PCSK9 serum levels were correlated with increased risk of MI in an sex- and age-adjusted analysis [53]. Accordingly, using the AMI rat model, Zhang et al. demonstrated that the concentration plasma of PCSK9 was significantly enhanced from 12 to 96 h at the acute stage of AMI in the rat model and verified by increased levels of liver mRNA [54]. Their results are consistent with genetic studies that have suggested a positive relationship between reduced risk of MI and a lower plasma level of PCSK9 [55,56,57].

Emerging studies have supported the connection between PCSK9, atherosclerosis, and MI. Growing evidence has also shown the significant effects of PCSK9 in lowering LDL-c plasma levels [49,58]. Interestingly, subsequent studies then found the ability of PCSK9 to induce atherosclerosis, independently from the LDL-c plasma levels [59,60]. PCSK9 was found to be a biomarker that can predict cardiovascular events, even in those patients with controlled LDL-c plasma levels [61]. Accordingly, collective studies have demonstrated that PCSK9 directly promotes atherosclerosis by being involved in atherosclerotic inflammation [16,17,18]. Furthermore, recent studies suggested a novel role for PCSK9 in promoting atherosclerosis through platelet activation, a key role during atherogenesis and in atherothrombosis-induced MI [35,36,62,63,64]. Notably, several clinical studies have demonstrated the antiplatelet effects of PCSK9 in patients with hypercholesterolemia and found it to be an effective and safe strategy for treating patients with uncontrolled hyperlipidemia and coronary artery disease [65,66].

## 5. PCSK9 Promotes Platelet Activation

In recent years, PCSK9 has been linked to platelet activation during atherosclerosis disease progression [35]. This indication was started by a cross-sectional study conducted by Li et al., who found a positive and independent association between plasma PCSK9 level and platelet count from a total of 330 stable coronary artery disease patients [67]. Their study was the first to give a hint about a link between high PCSK9 levels, platelets, atherosclerosis, and cardiovascular disorders [67]. Another study by Pastori et al. found a strong relationship between elevated PCSK9 and high urinary excretion of 11-dehydrothromboxane B2 (11-dh-TxB2), a stable metabolite of thromboxane A2, in patients at high risk of cardiovascular complication [68], suggesting a potential role of PCSK9 in regulating platelet activation. Accordingly, urine excretion of 11-dh-TxB2 is widely used as a predictive marker of MI or cardiovascular incidents in aspirin-treated patients [69]. The potential mechanism underlying the connection between urinary 11-dh-TxB2 and PCSK9 might lead to the possible involvement of cyclooxygenase (COX)-1, an essential enzyme for thromboxane A2; however, other mechanisms should be considered [68].

The PCSK9-REACT study (association of PCSK9 serum levels and platelet reactivity in patients with acute coronary syndrome treated with prasugrel or ticagrelor) by ATLANTIS-ACS (association between the antiplatelet drug efficacy/safety and platelet function in patients treated with novel platelet inhibitors due to an acute coronary syndrome) further highlighted the significant and direct relationship of higher PCSK9 levels and higher platelet reactivity [70]. Furthermore, their study demonstrated the association between elevated PCSK9 level and a higher incidence of atherothrombotic events, suggesting that PCSK9 can be used as a biomarker of clinical ischemic incidents and higher platelet activation, independently of other factors in acute coronary syndrome patients [70]. These clinical studies were further strengthened by an experimental study conducted by Camera et al., who used an animal model to investigate the effect of PCSK9 on platelet, activation, aggregation, and thrombosis [15]. The results indicated that the depletion of PCSK9 reduced the generation and stability of platelet function and arterial thrombus in mice [15]. In addition, platelet activation, which was assessed by the expression activated P-selectin and GP IIb/IIIa, and platelet–leukocyte aggregates, was decreased by 60% in the mutant mice (*PCSK9^−/−^)* compared with wild type mice (*PCSK9^+/+^)* [15]. Another in vivo study by Wang et al. found that eliminating PCSK9 had a protective effect on thrombosis, as evidenced by decreased leukocyte attachment on venous thrombosis, circulating lipid profile levels, and P-selectin levels in PCSK9-deficient mice [71].

Another interesting in vitro study was conducted by Petersen et al., who demonstrated that PCSK9 was stored and released by platelets in the presence of LDL [64]. The PCSK9-derived platelet was found to promote platelet aggregation, thrombus formation, monocyte migration, and monocyte differentiation into foam cells, all of which contributed to the occurrence of atherosclerosis progression-induced coronary artery disease [64]. Next, experimental studies were performed in an attempt to find a potential mechanism underlying PCSK9 and platelet activation in the progression of atherosclerosis. Cammisoto et al. began the investigation by performing a cross-sectional study complemented by an in vitro study to elucidate the molecular pathway involved in PCSK9 and platelet activation [72]. The results of their cross-sectional study on patients with atrial fibrillation not receiving antiplatelet drugs indicated that the plasma levels of PCSK9 are strongly and positively associated with oxidative stress markers and platelet activation, such as ROS, oxLDL, serum TxB2 formation, and P-selectin release [72]. Furthermore, the in vitro study demonstrated the involvement of CD36 and Nox2 activation-mediated ROS pathway as the underlying mechanism of platelet activation induction by PCSK9 [72]. Through co-immunoprecipitation analysis, they further demonstrated that PCSK9 binds to CD36, suggesting that PCSK9 activates platelets through direct binding with CD36 platelet receptors and activates the downstream pathway to induce platelet aggregation, including ROS derived from Nox2 activation [72].

A recent study by Qi et al. suggested a direct correlation between PCSK9 and platelet activation enhancement, which is in agreement with the previous study by Cammisoto et al. [36]. Using human recombinant PCSK9 in human platelets induced by ADP, thrombin, and collagen, they found that PCSK9 enhances platelet integrin αIIbβ3 activation, ATP release, P-selectin release from α-granules, and clot formation, resulting in platelet aggregation, microvascular obstruction, and eventually ischemic events that lead to MI [36]. They further found a direct interaction between PCSK9 and CD36 receptor in enhancing platelet activation by activating Src, ERK5, and JNK, increasing the ROS generation, and activating the p38/cPLA2/COX-1/TXA_2_ pathways [36]. Further in vivo studies also suggested that PCSK9 enhances thrombosis and that the effects are eliminated by aspirin, which restricts TbAx2 synthesis [36]. This indicates that aspirin can be used in patients with a high plasma level of PCSK9, complemented with PCSK9 inhibitors for the treatment or prevention of thrombotic complications [36]. They also further confirmed that PCSK9-induced platelet activation is dependent on CD36 platelet receptor, and unlike its interaction with LDLR, their finding additionally suggested that PCSK9 treatment did not alter the surface expression levels of CD36 [36].

These effects are not limited to CD36; the interaction between PCSK9 and LOX-1 in arterial tissues, cultured endothelial cells (ECs), and vascular smooth muscle cells also forms a potential mechanism for platelet activation [62]. Although the study by Cammisoto et al. could not verify the direct binding between PCSK9 and LOX-1, others have provided evidence about the positive feedback between PCSK9 and LOX-1, as well as CD36 and SARA, which leads to higher oxLDL uptake within platelet and subsequently, higher mitochondrial ROS generation [73,74]. Mitochondrial ROS generation is well known to mediate inflammation in generating foam cells, one of the key factors during both atherogenesis and atherothrombosis. The roles of PCSK9 in regulating the inflammation pathway in atherosclerosis-induced cardiovascular diseases have been well addressed in a previous review [17].

Hence, it is highly likely that PCSK9 inhibitors may inhibit both atherogenesis and atherothrombosis in hypercholesterolemia conditions, by disrupting CD36, LOX-1, and SARA expression [73,74]. Indeed, a study directed by Barale et al., who followed up a 12-month treatment of anti-PCSK9 monoclonal antibodies (alirocumab or evolocumab) in patients affected by primary hypercholesterolemia with statin and aspirin treatment (*n* = 24), demonstrated that patients benefit from anti-PCSK9 mAb treatment [75]. They showed a significant decrease in the LDL-c levels, and platelet reactivity, as well as enhanced platelet sensitivity to aspirin [75]. These data suggested that PCSK9 inhibitors could be used to reduce cardiovascular events in patients with hypercholesterolemia [35,75].

## 6. Discussion and Future Perspective

**Figure 1:**
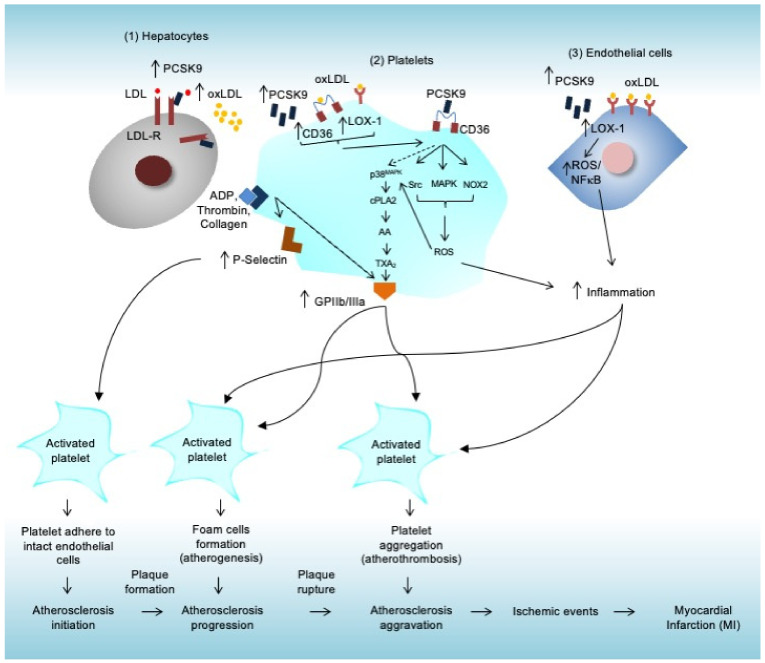
Graphical illustration of the PCSK9 pleiotropic effects on platelet activation-induced MI. (1) In hepatocytes, increased levels of PCSK9 lead to increased levels of LDL and oxLDL. (2) In platelets, supported by the binding of platelet with its agonists (ADP, collagen, and thrombin), an increased level of PCSK9 leads to an increased level of P-selectin and GPIIb/IIIa, which is important for platelet adhesion and activation; PCSK9 binds to CD36, which activates cPLA2 and thus activates the platelet coagulation signaling pathways via the p38MAPK pathway that promotes thrombus formation; PCSK9 and CD36 binding also activates Src-, MAPK-, and NOX2-mediated ROS generation that induces inflammation. In addition, increased levels of PCSK9 lead to increased levels of LOX-1 and CD36, which results in more uptake of oxLDL; thus, inducing the formation of foam cells. (3) In the endothelial cells, oxLDL binds to the LOX-1, which induces inflammation through the ROS and NFκB pathways and promotes plaque formation. Synergically, all of these processes activate the platelets that enhance and sustain the response for atherosclerosis development, from initiation, progression, to aggravation, which may lead to ischemic events, and finally MI occurrence. LDL: low-density lipoprotein; LDL-R: low-density lipoprotein receptor; oxLDL: oxidized LDL; LOX-1: lectin-like oxidized low-density lipoprotein receptor 1; CD36: cluster of differentiation 36; ADP: adenosine diphosphate; p38^MAPK^: p38 mitogen-activated protein kinase; cPLA2 = cytosolic phospholipase A2; AA: arachidonic acid; TXA2: thromboxane A2; NOX2: NADPH oxidase type 2; ROS: reactive oxygen species.

PCSK9 targets and enhances LDLR degradation, thus increasing LDL-c plasma levels. PCSK9 was recently found to directly bind with platelet CD36 and to then stimulate ROS generation-mediated inflammation, as well as activating coagulation signaling cascades. The cross-talk between PCSK9 and LOX-1 was also found to play a crucial role in mediating inflammation. Notably, inflammation also plays a crucial role in mediating atherogenesis and atherothrombosis, in which PCSK9 was found to enhance the inflammation process. Synergically, they initiate and promote the progression of atherogenesis and atherothrombosis in the arterial wall, which leads to clot formation, ischemic condition, and, eventually, MI (Figure 1). Further investigations are still required to explore the molecular machinery between PCSK9 and platelet activation, as well as the PCSK9 and CD36 protein–protein interaction. Since PCSK9 inhibitors, which are widely used as lipid-lowering agents, are currently available, continuous clinical studies are needed to further verify and evaluate their function for antiplatelet therapy. Using an experimental approach, finding the essential motif/domain for their interaction will also hopefully lead to the development of novel therapeutic drugs that selectively block their interactions, to inhibit platelet activation. Indeed, several antiplatelet and antithrombotic therapies have been established in the clinical field to treat MI; however, there is still much room for improvement. In addition, inhibiting PCSK9–CD36 interaction could be a promising strategy to develop more powerful drugs against platelet activation-induced MI.

## 7. Conclusions

Extensive research using experimental and clinical approaches has been directed toward exploring the functions of PCSK9 in cardiovascular diseases. PCSK9 was originally characterized by its effects on lipid metabolism and PCSK9 inhibitors, and found to be effective in treating patients with hyperlipidemia and for reducing the risk of advanced cardiovascular events, including MI. With its ability to control the lipid plasma level, the link between PCSK9 and atherosclerosis, a root cause for almost all cardiovascular diseases, was discovered. Interestingly, recent evidence showed the pleiotropic effect of PCSK9 ion atherosclerosis progression, beyond its lipid-lowering ability, potentially by being involved in platelet activation, a key event during the emergence of atherosclerosis initiation, atherogenesis, and atherothrombosis, which lead to the development of MI.

## References

[1] Roth G.A., Mensah G.A., Johnson C.O., Addolorato G., Ammirati E., Baddour L.M., Barengo N.C., Beaton A.Z., Benjamin E.J., Benziger C.P. (2020). Global Burden of Cardiovascular Diseases and Risk Factors, 1990-–2019: Update From the GBD 2019 Study. J. Am. Coll. Cardiol..

[2] Collaborators GDaI (2020). Global burden of 369 diseases and injuries in 204 countries and territories, 1990-2019: A systematic analysis for the Global Burden of Disease Study 2019. Lancet.

[3] Dunbar S.B., Khavjou O.A., Bakas T., Hunt G., Kirch R.A., Leib A.R., Morrison R.S., Poehler D.C., Roger V.L., Whitsel L.P. (2018). Projected Costs of Informal Caregiving for Cardiovascular Disease: 2015 to 2035: A Policy Statement from the American Heart Association. Circulation.

[4] World Health Organization (2021). Cardiovascular Diseases (CVDs). https://www.who.int/news-room/fact-sheets/detail/cardiovascular-diseases-(cvds).

[5] Stewart J., Manmathan G., Wilkinson P. (2017). Primary prevention of cardiovascular disease: A review of contemporary guidance and literature. JRSM Cardiovasc. Dis..

[6] Lopez E.O., Ballard B.D., Jan A. (2021). Cardiovascular Disease [Updated 2021 Aug 11]. StatPearls [Internet].

[7] Arnett D.K., Blumenthal R.S., Albert M.A., Buroker A.B., Goldberger Z.D., Hahn E.J., Himmelfarb C.D., Khera A., Lloyd-Jones D., McEvoy J.W. (2019). 2019 ACC/AHA guideline on the primary prevention of cardiovascular disease: Executive summary: A report of the American College of Cardiology/American Heart Association Task Force on Clinical Practice Guidelines. Circulation.

[8] Knuuti J., Wijns W., Saraste A., Capodanno D., Barbato E., Funck-Brentano C., Prescott E., Storey R.F., Deaton C., Cuisset T. (2020). 2019 ESC Guidelines for the diagnosis and management of chronic coronary syndromes. Eur. Heart J..

[9] Seidah N.G., Benjannet S., Wickham L., Marcinkiewicz J., Jasmin S.B., Stifani S., Basak A., Prat A., Chrétien M. (2003). The secretory proprotein convertase neural apoptosis-regulated convertase 1 (NARC-1): Liver regeneration and neuronal differentiation. Proc. Natl. Acad. Sci. USA.

[10] Seidah N.G. (2008). PCSK9 as a therapeutic target of dyslipidemia. Expert Opin. Ther. Targets.

[11] Seidah N.G., Awan Z., Chrétien M., Mbikay M. (2014). PCSK9: A key modulator of cardiovascular health. Circ. Res..

[12] Abifadel M., Varret M., Rabès J.-P., Allard D., Ouguerram K., Devillers M., Cruaud C., Benjannet S., Wickham L., Erlich D. (2003). Mutations in PCSK9 cause autosomal dominant hypercholesterolemia. Nat. Genet..

[13] Melendez Q.M., Krishnaji S.T., Wooten C.J., Lopez D. (2017). Hypercholesterolemia: The role of PCSK9. Arch. Biochem. Biophys..

[14] Peterson A.S., Fong L.G., Young S.G. (2008). PCSK9 function and physiology. J. Lipid. Res..

[15] Camera M., Rossetti L., Barbieri S.S., Zanotti I., Canciani B., Trabattoni D., Ruscica M., Tremoli E., Ferri N. (2018). PCSK9 as a Positive Modulator of Platelet Activation. J. Am. Coll. Cardiol..

[16] Hachem A., Hariri E., Saoud P., Lteif C., Lteif L., Welty F. (2017). The Role of Proprotein Convertase Subtilisin/Kexin Type 9 (PCSK9) in Cardiovascular Homeostasis: A Non-Systematic Literature Review. Curr. Cardiol. Rev..

[17] Momtazi A.A., Sabouri-Rad S., Gotto A.M., Pirro M., Banach M., Awan Z., E Barreto G., Sahebkar A. (2019). PCSK9 and inflammation: A review of experimental and clinical evidence. Eur. Hear. J. Cardiovasc. Pharmacother..

[18] Guo Y., Yan B., Tai S., Zhou S., Zheng X.L. (2021). PCSK9: Associated with cardiac diseases and their risk factors?. Arch Biochem. Biophys..

[19] Thygesen K., Alpert J.S., Jaffe A.S., Chaitman B.R., Bax J.J., Morrow D.A., White H.D. (2018). Fourth Universal Definition of Myocardial Infarction (2018). J. Am. Coll. Cardiol..

[20] Mechanic O.J., Gavin M., Grossman S.A. (2022). Acute Myocardial Infarction.

[21] Lu L., Liu M., Sun R., Zheng Y., Zhang P. (2015). Myocardial Infarction: Symptoms and Treatments. Cell Biophys..

[22] Collet J.P., Thiele H., Barbato E., Barthélémy O., Bauersachs J., Bhatt D.L., Dendale P., Dorobantu M., Edvardsen T., Folliguet T. (2021). 2020 ESC Guidelines for the management of acute coronary syndromes in patients presenting without persistent ST-segment elevation. Eur. Heart J..

[23] Ibanez B., James S., Agewall S., Antunes M.J., Bucciarelli-Ducci C., Bueno H., Caforio A.L.P., Crea F., Goudevenos J.A., Halvorsen S. (2018). 2017 ESC Guidelines for the management of acute myocardial infarction in patients presenting with ST-segment elevation: The Task Force for the management of acute myocardial infarction in patients presenting with ST-segment elevation of the European Society of Cardiology (ESC). Eur. Heart J..

[24] Badimon L., Padró T., Vilahur G. (2012). Atherosclerosis, platelets and thrombosis in acute ischaemic heart disease. Eur. Heart J. Acute Cardiovasc. Care.

[25] Hajar R. (2017). Risk factors for coronary artery disease: Historical perspectives. Hear. Views.

[26] Linton M.F., Yancey P.G., Davies S.S., Jerome W.G., Linton E.F., Song W.L., Doran A.C., Vickers K.C., Feingold K.R., Anawalt B., Boyce A., Chrousos G., de Herder W.W., Dhatariya K. (2000). The Role of Lipids and Lipoproteins in Atherosclerosis. Endotext.

[27] Kanuri S.H., Mehta J.L. (2019). Role of Ox-LDL and LOX-1 in Atherogenesis. Curr. Med. Chem..

[28] Collot-Teixeira S., Martin J., McDermott-Roe C., Poston R., McGregor J.L. (2007). CD36 and macrophages in atherosclerosis. Cardiovasc. Res..

[29] Marwali M.R., Hu C.-P., Mohandas B., Dandapat A., Deonikar P., Chen J., Cawich I., Sawamura T., Kavdia M., Mehta J.L. (2007). Modulation of ADP-Induced Platelet Activation by Aspirin and Pravastatin: Role of Lectin-Like Oxidized Low-Density Lipoprotein Receptor-1, Nitric Oxide, Oxidative Stress, and Inside-Out Integrin Signaling. J. Pharmacol. Exp. Ther..

[30] Massberg S., Brand K., Gruner S., Page S., Muller E., Muller I., Bergmeier W., Richter T., Lorenz M., Konrad I. (2002). A Critical Role of Platelet Adhesion in the Initiation of Atherosclerotic Lesion Formation. J. Exp. Med..

[31] Lindemann S., Krämer B., Seizer P., Gawaz M. (2007). Platelets, inflammation and atherosclerosis. J. Thromb. Haemost..

[32] Pamukcu B., Lip G.Y., Shantsila E. (2011). The nuclear factor—Kappa B pathway in atherosclerosis: A potential therapeutic target for atherothrombotic vascular disease. Thromb. Res..

[33] Yang M., Cooley B.C., Li W., Chen Y., Vasquez-Vivar J., Scoggins N.O., Cameron S.J., Morrell C.N., Silverstein R.L. (2017). Platelet CD36 promotes thrombosis by activating redox sensor ERK5 in hyperlipidemic conditions. Blood.

[34] Lahav J., Jurk K., Hess O., Barnes M.J., Farndale R.W., Luboshitz J., Kehrel B.E. (2002). Sustained integrin ligation involves extracellular free sulfhydryls and enzymatically catalyzed disulfide exchange. Blood.

[35] Barale C., Melchionda E., Morotti A., Russo I. (2021). PCSK9 Biology and Its Role in Atherothrombosis. Int. J. Mol. Sci..

[36] Qi Z., Hu L., Zhang J., Yang W., Liu X., Jia D., Yao Z., Chang L., Pan G., Zhong H. (2021). PCSK9 (Proprotein Convertase Subtilisin/Kexin 9) Enhances Platelet Activation, Thrombosis, and Myocardial Infarct Expansion by Binding to Platelet CD36. Circulation.

[37] Palasubramaniam J., Wang X., Peter K. (2019). Myocardial Infarction-From Atherosclerosis to Thrombosis. Arterioscler. Thromb. Vasc. Biol..

[38] Chrétien M. (2012). My road to Damascus: How I converted to the prohormone theory and the proprotein convertases. Biochem. Cell Biol..

[39] Steiner D.F. (2011). On the Discovery of Precursor Processing. Program. Necrosis.

[40] Seidah N.G. (2017). The PCSK9 revolution and the potential of PCSK9-based therapies to reduce LDL-cholesterol. Glob. Cardiol. Sci. Pract..

[41] Maxwell K.N., Soccio R.E., Duncan E.M., Sehayek E., Breslow J.L. (2003). Novel putative SREBP and LXR target genes identified by microarray analysis in liver of cholesterol-fed mice. J. Lipid Res..

[42] Dubuc G., Chamberland A., Wassef H., Davignon J., Seidah N.G., Bernier L., Prat A. (2004). Statins Upregulate *PCSK9*, the Gene Encoding the Proprotein Convertase Neural Apoptosis-Regulated Convertase-1 Implicated in Familial Hypercholesterolemia. Arter. Thromb. Vasc. Biol..

[43] Abifadel M., Rabès J.-P., Devillers M., Munnich A., Erlich D., Junien C., Varret M., Boileau C. (2009). Mutations and polymorphisms in the proprotein convertase subtilisin kexin 9 (PCSK9) gene in cholesterol metabolism and disease. Hum. Mutat..

[44] Horton J.D., Cohen J.C., Hobbs H.H. (2007). Molecular biology of PCSK9: Its role in LDL metabolism. Trends Biochem. Sci..

[45] Benjannet S., Rhainds D., Essalmani R., Mayne J., Wickham L., Jin W., Assenlin M.-C., Hamelin J., Varret M., Allard D. (2004). NARC-1/PCSK9 and its natural mutants: Zymogen cleavage and effects on the low density lipoprotein (LDL) receptor and LDL cholesterol. J. Biol. Chem..

[46] Zhang D.-W., Lagace T.A., Garuti R., Zhao Z., McDonald M., Horton J.D., Cohen J.C., Hobbs H.H. (2007). Binding of Proprotein Convertase Subtilisin/Kexin Type 9 to Epidermal Growth Factor-like Repeat A of Low Density Lipoprotein Receptor Decreases Receptor Recycling and Increases Degradation. J. Biol. Chem..

[47] Denis M., Marcinkiewicz J., Zaid A., Gauthier D., Poirier S., Lazure C., Seidah N.G., Prat A. (2012). Gene Inactivation of Proprotein Convertase Subtilisin/Kexin Type 9 Reduces Atherosclerosis in Mice. Circulation.

[48] Rashid S., Curtis D.E., Garuti R., Anderson N.N., Bashmakov Y., Ho Y.K., Hammer R.E., Moon Y.-A., Horton J.D. (2005). Decreased plasma cholesterol and hypersensitivity to statins in mice lacking Pcsk9. Proc. Natl. Acad. Sci. USA.

[49] Paton D. (2016). PCSK9 inhibitors: Monoclonal antibodies for the treatment of hypercholesterolemia. Drugs Today.

[50] Yusuf S., Hawken S., Ôunpuu S., Dans T., Avezum A., Lanas F., McQueen M., Budaj A., Pais P., Varigos J. (2004). Effect of potentially modifiable risk factors associated with myocardial infarction in 52 countries (the INTERHEART study): Case-control study. Lancet.

[51] Balci B. (2012). The Modification of Serum Lipids after Acute Coronary Syndrome and Importance in Clinical Practice. Curr. Cardiol. Rev..

[52] Almontashiri N.A.M., Vilmundarson R.O., Ghasemzadeh N., Dandona S., Roberts R., Quyyumi A.A., Chen H.-H., Stewart A.F.R. (2014). Plasma PCSK9 Levels Are Elevated with Acute Myocardial Infarction in Two Independent Retrospective Angiographic Studies. PLoS ONE.

[53] Laugsand L.E., Åsvold B.O., Vatten L.J., Janszky I., Platou C.G., Michelsen A.E., Damås J.K., Aukrust P., Ueland T. (2016). Circulating PCSK9 and Risk of Myocardial Infarction. The HUNT Study in Norway. JACC Basic Transl. Sci..

[54] Zhang Y., Liu J., Li S., Xu R.-X., Sun J., Tang Y., Li J.-J. (2014). Proprotein convertase subtilisin/kexin type 9 expression is transiently up-regulated in the acute period of myocardial infarction in rat. BMC Cardiovasc. Disord..

[55] Benn M., Nordestgaard B.G., Grande P., Schnohr P., Tybjærg-Hansen A. (2010). PCSK9R46L, Low-Density Lipoprotein Cholesterol Levels, and Risk of Ischemic Heart Disease: 3 Independent Studies and Meta-Analyses. J. Am. Coll. Cardiol..

[56] Kathiresan S. (2008). A PCSK9 missense variant associated with a reduced risk of early-onset myocardial infarction. N. Engl. J. Med..

[57] Cohen J.C., Boerwinkle E., Mosley T.H., Hobbs H.H. (2006). Sequence Variations inPCSK9,Low LDL, and Protection against Coronary Heart Disease. N. Engl. J. Med..

[58] Farnier M. (2014). PCSK9: From discovery to therapeutic applications. Arch. Cardiovasc. Dis..

[59] Cheng J.M., Oemrawsingh R.M., Garcia-Garcia H.M., Boersma E., van Geuns R.-J., Serruys P.W., Kardys I., Akkerhuis K.M. (2016). PCSK9 in relation to coronary plaque inflammation: Results of the ATHEROREMO-IVUS study. Atherosclerosis.

[60] Giunzioni I., Tavori H., Covarrubias R., Major A.S., Ding L., Zhang Y., DeVay R.M., Hong L., Fan D., Predazzi I.M. (2016). Local effects of human PCSK9 on the atherosclerotic lesion. J. Pathol..

[61] Shapiro M.D., Fazio S. (2017). PCSK9 and Atherosclerosis—Lipids and Beyond. J. Atheroscler. Thromb..

[62] Gurbel P.A., Navarese E.P., Tantry U.S. (2017). Exploration of PCSK9 as a Cardiovascular Risk Factor: Is There a Link to the Platelet?. J. Am. Coll. Cardiol..

[63] Paciullo F., Momi S., Gresele P. (2019). PCSK9 in Haemostasis and Thrombosis: Possible Pleiotropic Effects of PCSK9 Inhibitors in Cardiovascular Prevention. Thromb. Haemost..

[64] Petersen-Uribe Á, Kremser M., Rohlfing A.-K., Castor T., Kolb K., Dicenta V., Emschermann F., Li B., Borst O., Rath D. (2021). Platelet-Derived PCSK9 Is Associated with LDL Metabolism and Modulates Atherothrombotic Mechanisms in Coronary Artery Disease. Int. J. Mol. Sci..

[65] Pęczek P., Leśniewski M., Mazurek T., Szarpak L., Filipiak K., Gąsecka A. (2021). Antiplatelet Effects of PCSK9 Inhibitors in Primary Hypercholesterolemia. Life.

[66] Rosenson R.S., Hegele R.A., Fazio S., Cannon C.P. (2018). The Evolving Future of PCSK9 Inhibitors. J. Am. Coll. Cardiol..

[67] Li S., Zhu C.-G., Guo Y.-L., Xu R.-X., Zhang Y., Sun J., Li J.-J. (2015). The Relationship between the Plasma PCSK9 Levels and Platelet Indices in Patients with Stable Coronary Artery Disease. J. Atheroscler. Thromb..

[68] Pastori D., Nocella C., Farcomeni A., Bartimoccia S., Santulli M., Vasaturo F., Carnevale R., Menichelli D., Violi F., Pignatelli P. (2017). Relationship of PCSK9 and Urinary Thromboxane Excretion to Cardiovascular Events in Patients with Atrial Fibrillation. J. Am. Coll. Cardiol..

[69] Eikelboom J.W., Hankey G.J., Thom J., Bhatt D.L., Steg P.G., Montalescot G., Johnston S.C., Steinhubl S.R., Mak K.-H., Easton J.D. (2009). Incomplete Inhibition of Thromboxane Biosynthesis by Acetylsalicylic Acid: Determinants and Effect on Cardiovascular Risk. Circulation.

[70] Navarese E.P., Kołodziejczak M., Winter M.-P., Alimohammadi A., Lang I.M., Buffon A., Lip G.Y., Siller-Matula J.M. (2017). Association of PCSK9 with platelet reactivity in patients with acute coronary syndrome treated with prasugrel or ticagrelor: The PCSK9-REACT study. Int. J. Cardiol..

[71] Wang H., Wang Q., Wang J., Guo C., Kleiman K., Meng H., Knight J.S., Eitzman D.T. (2017). Proprotein convertase subtilisin/kexin type 9 (PCSK9) Deficiency is Protective Against Venous Thrombosis in Mice. Sci. Rep..

[72] Cammisotto V., Pastori D., Nocella C., Bartimoccia S., Castellani V., Marchese C., Scavalli A.S., Ettorre E., Viceconte N., Violi F. (2020). PCSK9 Regulates Nox2-Mediated Platelet Activation via CD36 Receptor in Patients with Atrial Fibrillation. Antioxidants.

[73] Ding Z., Liu S., Wang X., Deng X., Fan Y., Shahanawaz J., Reis R.J.S., Varughese K.I., Sawamura T., Mehta J.L. (2015). Cross-talk between LOX-1 and PCSK9 in vascular tissues. Cardiovasc. Res..

[74] Ding Z., Liu S., Wang X., Theus S., Deng X., Fan Y., Zhou S., Mehta J.L. (2018). PCSK9 regulates expression of scavenger receptors and ox-LDL uptake in macrophages. Cardiovasc. Res..

[75] Barale C., Bonomo K., Frascaroli C., Morotti A., Guerrasio A., Cavalot F., Russo I. (2020). Platelet function and activation markers in primary hypercholesterolemia treated with anti-PCSK9 monoclonal antibody: A 12-month follow-up. Nutr. Metab. Cardiovasc. Dis..

